# Interferon alpha inhibits spinal cord synaptic and nociceptive transmission via neuronal-glial interactions

**DOI:** 10.1038/srep34356

**Published:** 2016-09-27

**Authors:** Chien-Cheng Liu, Yong-Jing Gao, Hao Luo, Temugin Berta, Zhen-Zhong Xu, Ru-Rong Ji, Ping-Heng Tan

**Affiliations:** 1 Department of Anesthesiology, E-Da Hospital, School of Medicine, I-Shou University, Kaohsiung, Taiwan; 2 Sensory Plasticity Laboratory, Pain Research Center, Department of Anesthesiology, Brigham and Women’s Hospital and Harvard Medical School, Boston, Massachusetts 02115, USA; 3 Pain Research Laboratory, Institute of Nautical Medicine, Jiangsu Key Laboratory of Inflammation and Molecular Drug Target, Nantong University, Nantong, Jiangsu 226019, China; 4 Department of Anesthesiology, Duke University Medical Center, Durham, North Carolina 27710, USA.

## Abstract

It is well known that interferons (IFNs), such as type-I IFN (IFN-α) and type-II IFN (IFN-γ) are produced by immune cells to elicit antiviral effects. IFNs are also produced by glial cells in the CNS to regulate brain functions. As a proinflammatory cytokine, IFN-γ drives neuropathic pain by inducing microglial activation in the spinal cord. However, little is known about the role of IFN-α in regulating pain sensitivity and synaptic transmission. Strikingly, we found that IFN-α/β receptor (type-I IFN receptor) was expressed by primary afferent terminals in the superficial dorsal horn that co-expressed the neuropeptide CGRP. In the spinal cord IFN-α was primarily expressed by astrocytes. Perfusion of spinal cord slices with IFN-α suppressed excitatory synaptic transmission by reducing the frequency of spontaneous excitatory postsynaptic current (sEPSCs). IFN-α also inhibited nociceptive transmission by reducing capsaicin-induced internalization of NK-1 and phosphorylation of extracellular signal-regulated kinase (ERK) in superficial dorsal horn neurons. Finally, spinal (intrathecal) administration of IFN-α reduced inflammatory pain and increased pain threshold in naïve rats, whereas removal of endogenous IFN-α by a neutralizing antibody induced hyperalgesia. Our findings suggest a new form of neuronal-glial interaction by which IFN-α, produced by astrocytes, inhibits nociceptive transmission in the spinal cord.

Interferons (IFNs) were discovered as natural antiviral substances induced during viral infection and were initially named for their ability to “interfere” with viral replication, slow cell proliferation, and modulate immunity. The IFN family is divided into two groups: Type-I IFNs consist of IFN-α and IFN-β, two of the major members, as well as IFN-ω, IFN-τ, IFN-δ, IFN-κ, and IFN-ε; whereas type-II IFN only includes IFN-γ^1^,^2^. Type-I IFNs share a common receptor and exhibit similar biological activities^2^. IFNs have been used in various clinical settings. For example, type-I IFNs are used to treat hepatitis-B/C, leukemia, and multiple sclerosis^3^,^4^. IFNs are continuously produced *in vivo* by immune cells such as macrophages, monocytes, and T lymphocytes for maintaining physiological function^5^,^6^. A weak IFN signaling is important to maintain the homeostasis of the immune system^7^. Type-I and II IFNs are also produced by glia such as astrocytes in the central nervous system^3^,^8^,^9^,^10^. IFNs modulate neurophysiological activities of brain regions involving in temperature control and food intake^3^.

Recent progress in pain research has demonstrated a critical role of glial cells such as microglia and astrocytes in the pathogenesis of pain via producing inflammatory mediators to mediate neuronal-glial interactions in the spinal cord and supraspinal region^11^,^12^,^13^,^14^,^15^,^16^,^17^,^18^. Several lines of evidence indicate that IFN-γ might mediate neuronal-glial interactions in the spinal cord in neuropathic pain. First, IFN-γ is produced by spinal cord astrocytes, microglia and infiltrating T cells^8^,^19^. Second, IFN-γ drives neuropathic pain by activating microglia that express IFN-γ receptor^20^. Third, IFN-γ can directly increase excitatory synaptic transmission in the spinal cord^21^.

In contrast to well-documented pronociceptive role of IFN-γ, it is virtually unknown whether and how IFN-α regulates pain sensitivity in the spinal cord. In this study, we investigated the expression of IFN-α and its type-I IFN receptor (IFN-α/βR) in the spinal cord and explored the role of IFN-α in modulating nociceptive synaptic transmission in the spinal cord. Our data showed that IFN-α acts as an endogenous pain suppressor via a novel form of neuronal-glial interaction.

## Results

### IFN-α receptors are expressed in spinal cord terminals and primary sensory neurons

**Figure 1:**
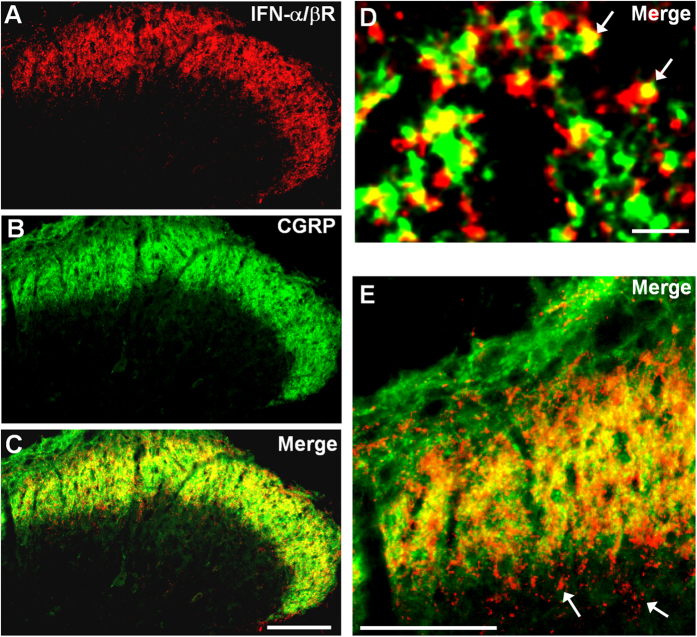
Expression of IFN-α receptors in the spinal cord dorsal horn. (**A–C**) Double staining of Type I-IFN receptor (IFN-α/βR) and CGRP in the superficial dorsal horn. Scale, 100 μm. (**D,E**) High magnification images showing colocalization of IFN-α/βR and CGRP in primary afferent terminals in the superficial dorsal horn (laminae I-IIo). Arrows in (**D**) indicate double-labeled terminals. Arrows in E show IFN-α/βR labeling in inner lamina II (IIi). Scales, 3 μm (**D**) and 100 μm (**E**).

To determine the role of spinal IFN-α in pain modulation, we first investigated the expression of type-I IFN receptor (IFN-α/βR) in the spinal cord. Remarkably, IFN-α/βR expression was restricted to the superficial dorsal horn (laminae I-II) in the spinal cord, where nociceptive primary afferents (C/Aδ) terminate Fig. 1A). Double staining further demonstrated that IFN-α/βR was co-localized with the neuropeptide calcitonin gene-related peptide (CGRP) (Fig. 1B–D) in axonal terminals in the lamina I-IIo. Further analysis in higher magnification image showed additional IFN-α/βR staining in axonal terminals in the inner lamina II (IIi, Fig. 1E), suggesting that non-peptidergic fibers may also express IFN-α/βR. Together, these results imply that IFN-α/βR is predominantly expressed on C-fibers on the superficial dorsal horn.

**Figure 2:**
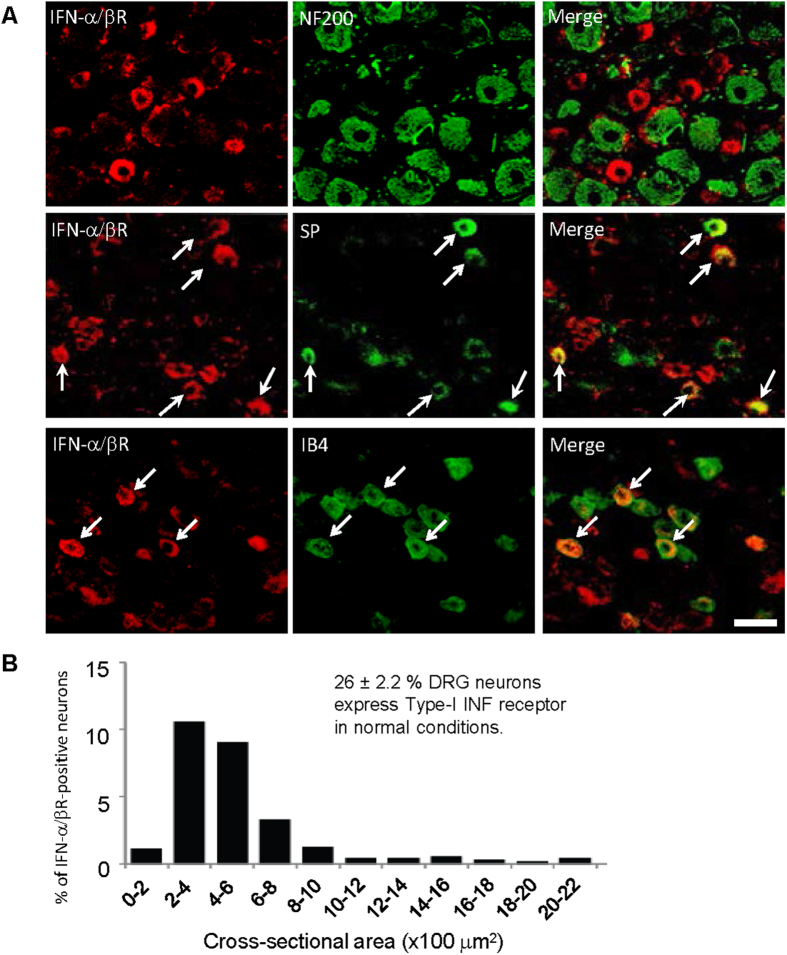
Expression of IFN-α receptors in primary sensory neurons in the DRG. (**A**) Double staining showing colocalization of IFN-α/βR with substance P and IB4 but not with NF200 in the DRG. Arrows indicate double-labeled neurons. Scale, 50 μm. (**B**) Size frequency distribution of IFN-α/βR-positive neurons in the DRG. Twelve DRG sections from 4 animals were included for quantification. Note that 26% DRG neurons express IFN-α/βR in normal conditions. Most IFN-α/βR-positive neurons have cross-sectional areas of 200–600 μm^2^.

To define whether IFN-α/βR in the spinal cord is originated from primary afferent neurons, we examined IFN-α/βR expression the dorsal root ganglion (DRG). In DRG sections, IFN-α/βR was expressed by small-sized neurons that are negative for NF-200, a marker for myelinated large-sized A fiber neurons. Thus, IFN-α/βR is mainly present in C-fiber neurons (Fig. 2A). Size frequency analysis revealed that most IFN-α/βR-positive neurons had the size of small neurons, with a cross section area of 200–600 μm^2^ (Fig. 2B). Further analysis showed that 26% of DRG neurons were positive for IFN-α/βR. In parallel with spinal cord staining, IFN-α/βR-positive neurons co-expressed the neuropeptide substance P (Fig. 2A). In addition, non-peptidergic neurons (IB4+) expressed IFN-α/βR (Fig. 2A). The unique distribution patterns of type-I receptors in the DRG and spinal cord strongly suggest a role of the receptors in nociception.

### IFN-α inhibits spinal cord synaptic transmission

**Figure 3:**
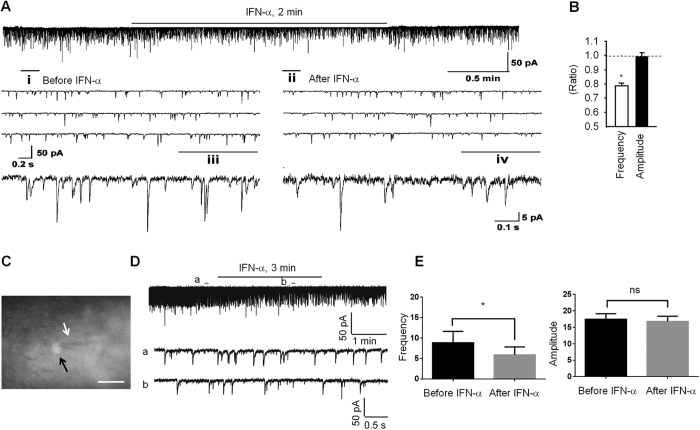
IFN-α inhibits excitatory synaptic transmission in IIo neurons of rat and mouse spinal cord. (**A**) Patch clamp recording in spinal cord slice (*ex vivo*) shows an inhibition of the frequency but not the amplitude of spontaneous excitatory postsynaptic currents (sEPSCs) in lamina II neurons after superfusion of IFN-α (rat, 25 ng/ml, 2 min). i and ii are enlarged recordings before and after IFN-α application. iii and iv are further enlargements of i and ii, respectively. (**B**) Frequency and amplitude of sEPSCs, expressed as ratio of baseline. 7 out of 9 recorded neurons respond to IFN-α. *P < 0.05, *n* = 7 neurons/group. The comparison was made between pre-treatment baseline and post-treatment in the same neurons using two-tailed paired student’s t-test. (**C**) Mouse spinal cord slice image showing a recording electrode (white arrow) in a SOM^+^ neuron (black arrow). Scale, 20 μm. (**D)** Traces of sEPSCs in mouse spinal cord slice before and after the IFN-α treatment (mouse, 50 Units/ml). (**E**) Frequency and amplitude of sEPSCs in mouse spinal cord slice. *P < 0.05, two-tailed paired student’s test, *n* = 6 neurons/group. ns, not significant. All the data were mean ± S.E.M.

Since IFN-α/βR is localized in axonal terminals in the spinal cord, we hypothesized that IFN-α modulates neurotransmitter release and synaptic transmission in the spinal cord. To test this hypothesis, we used patch clamp technique to record spontaneous excitatory synaptic currents (sEPSCs) in lamina IIo nociceptive neurons in isolated spinal cord slices from rats. Application of IFN-α to spinal cord slices (25 ng/ml) significantly reduced the frequency of sEPSCs without changing the amplitude of sEPSCs (P < 0.05, paired two-tailed t-test) (Fig. 3A,B). Since 1) sEPSC is mediated by glutamate receptors (AMPA/Kainate receptors) and 2) frequency change of sEPSC is caused by presynaptic mechanism^22^,^23^,^24^, our results suggest that IFN-α inhibits excitatory synaptic transmission by suppressing glutamate release from presynaptic terminals.

Spinal cord lamina IIo neurons are predominantly excitatory neurons^25^ and form a nociceptive circuit: they receive input from C-fiber afferents and send output to lamina I projection neurons^26^. These excitatory interneurons also express somatostatin^27^. We also recorded from Somatostatin-positive (SOM^+^) neurons in lamina IIo from transgenic mice showing SOM expression with tdTomato, an exceptionally bright red fluorescent protein. Consistently, mouse IFN-α (50 units/ml) produced a marked inhibition of sEPSC frequency in SOM^+^ lamina IIo neurons of mouse spinal cord slices (P = 0.0219, paired two-tailed t-test, t = 3.281, n = 6 neurons/group) (Fig. 3C–E). This result suggests that IFN-α can suppress excitatory synaptic transmission in excitatory neurons. Since sEPSC can be completely blocked by the AMPA receptor antagonist CNQX and the frequency changes of sEPSC are likely to be mediated by presynaptic mechanisms^28^, we conclude that IFN-α inhibits nociceptive transmission via suppressing glutamate release from C-fiber afferents in the superficial dorsal horn.

### IFN-α inhibits nociceptive signaling transduction in the spinal cord

**Figure 4:**
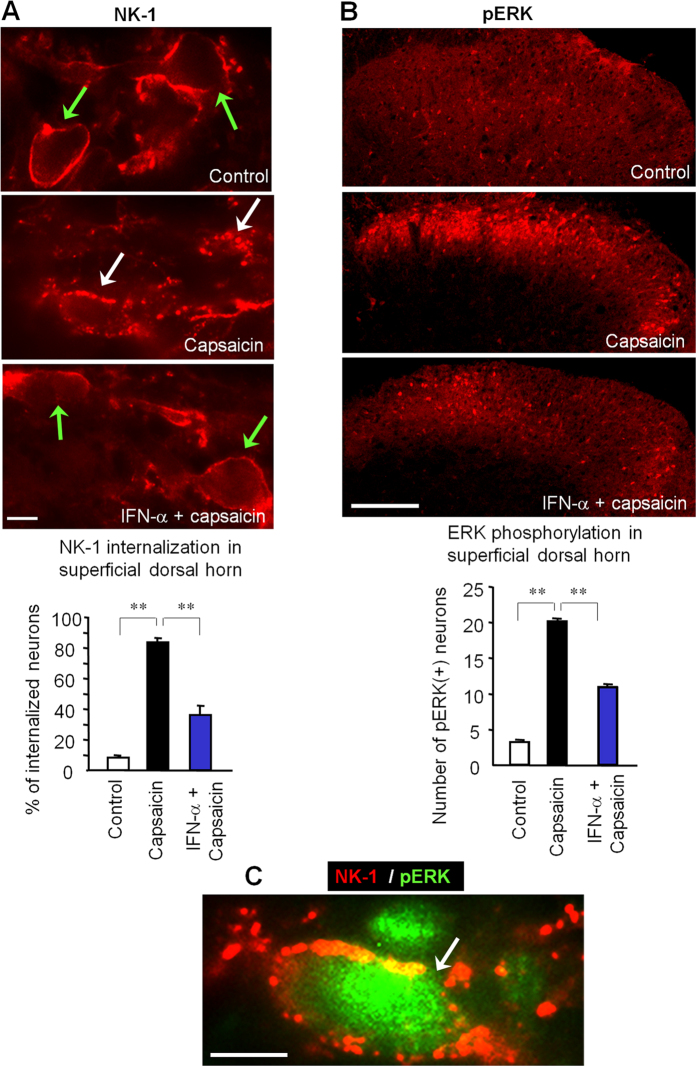
IFN-α inhibits nociceptive signal transduction in the spinal cord. (**A**) Intraplantar capsaicin (75 μg, 5 min) induces NK-1 internalization in superficial dorsal horn neurons, which is suppressed by IFN-α (i.t., 100 ng). Green and white arrows indicate surface-expressed and internalized NK-1 receptors, respectively. Scales, 10 μm. Low panel, Percentage of NK-1-positive neurons with receptor internalization in laminae I-II. **P < 0.01, one-way ANOVA, *n* = 4 rats/group. Scale, 10 μm. (**B**) Bath application of capsaicin (3 mM, 5 min) to spinal cord slices induces robust ERK phosphorylation (pERK), which is abolished by IFN-α (2.5 ng/ml). Scale, 100 μm. Low panel, number of pERK (+) neurons in superficial dorsal horn. **P < 0.01, one-way ANOVA, *n* = 4 slices from separate rats. (**C**) Double staining of NK-1 and pERK shows co-localization in a lamina I neuron following intraplantar capsaicin (75 μg, 5 min). Scale, 100 μm. All data were mean ± S.E.M.

To further support the idea that IFN-α inhibits nociceptive transmission in the spinal cord, we examined the release of the neuropeptide substance P, an important neurotransmitter for pain. Noxious stimulation-induced substance P release is measured by internalization of its NK-1 receptors in dorsal horn neurons^29^,^30^. NK-1 was normally expressed on cell surface in the superficial dorsal horn especially in lamina I, and only 10% NK-1-positive neurons showed notable NK-1 expression in endosomes (Fig. 4A). Intraplantar injection of capsaicin elicited marked NK-1 internalization: more than 80% NK-1-positve neurons exhibited NK-1 labeling in endosome-like structures after 5 min (Fig. 4A). Notably, intrathecal administration of IFN-α (100 ng), 30 min prior to capsaicin injection, significantly suppressed capsaicin-induced NK-1 internalization (Fig. 4A, P < 0.01, n = 4).

Phosphorylation of extracellular signal-regulated kinase (pERK) is regarded as a molecular marker for the activation and sensitization of spinal nociceptive neurons^31^,^32^,^33^. As we previously reported^34^, perfusion of spinal cord slices with capsaicin (3 μM) evoked robust pERK induction in superficial dorsal horn neurons in the laminae I-II. Of note capsaicin-induced pERK expression was suppressed by IFN-α perfusion (25 ng/ml, Fig. 4B). Interestingly, following capsaicin stimulation *in vivo*, pERK was also induced in the same neurons showing NK-1 internalization (Fig. 4C), suggesting these nociceptive-specific signaling evens might occur in NK-1 expressing projection neurons. However, we do not exclude the possibility that in chronic pain conditions, pERK can also be induced in non-neuronal cells such as microglia and astrocytes^35^,^36^.

### IFN-α is expressed in spinal cord astrocytes

**Figure 5:**
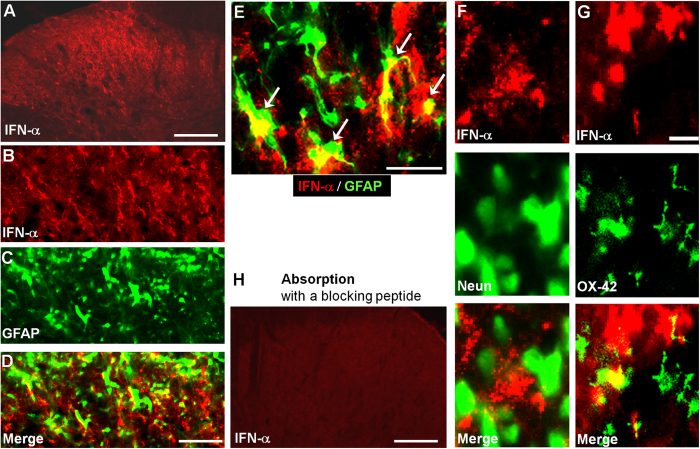
IFN-α expression in the spinal cord dorsal horn. (**A**) Immunohistochemistry showing IFN-α-expression in the spinal cord dorsal horn. Scale, 100 μm. (**B–D**) Double staining of IFN-α (**B**) and GFAP (**C**) in the superficial spinal cord dorsal horn. D is the merge of B and C. Scales, 50 μm. (**E**) High magnification image showing double staining of IFN-α and GFAP in the superficial spinal cord dorsal horn. Arrows indicate double-labeled astrocytes. Arrows indicate double-labeled cells. Scales, 25 μm. **(F,G)** Double staining of IFN-α with the neuronal marker NeuN (**F**) and microglial marker OX-42 (**G**) in the superficial spinal cord dorsal horn. Scales, 10 μm. **(H)** Absence of IFN-α immunostaining in the dorsal horn after absorption with a blocking peptide. Scale, 100 μm.

Immunofluorescence showed IFN-α immunoreactivity in the spinal cord dorsal horn (Fig. 5A). Double immunofluorescence revealed that IFN-α was predominantly expressed in spinal cord astrocytes expressing GFAP (Fig. 5B–E). IFN-α was not found in neurons (NeuN+) in the dorsal horn (Fig. 5F). Few microglial cells (OX-42+) also expressed IFN-α (Fig. 5G). IFN-α immunostaining was lost after absorption of the primary antibody with a specific blocking peptide (Fig. 5H) or after omission of primary antibody (data not shown), supporting the specificity of the staining.

**Figure 6:**
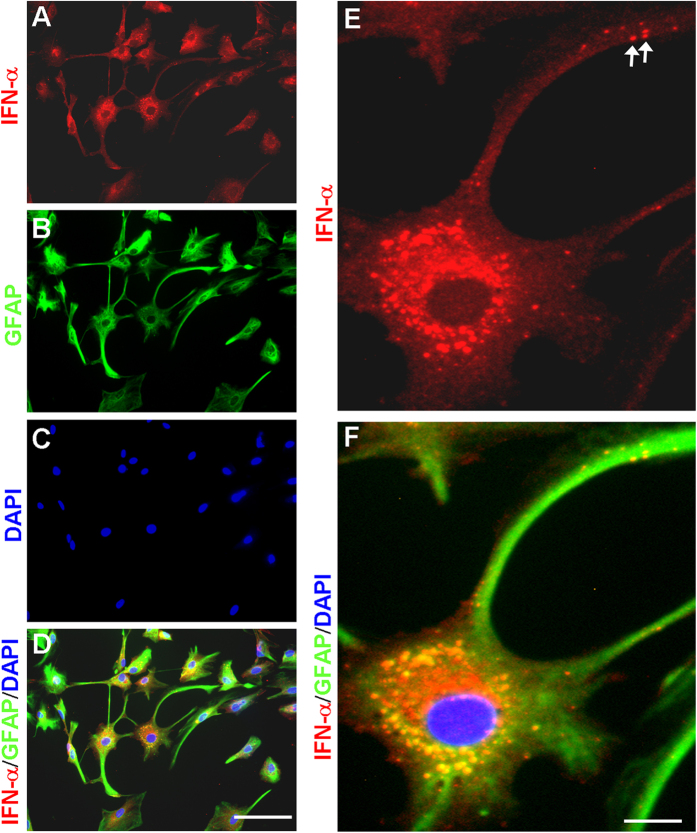
IFN-α expression in cultured astrocytes. (**A–D**) Triple staining of IFN-α (**A**), GFAP (**B**), and nucleus marker DAPI (**C**) in astrocytes. D is the merge of A-C. Scales, 30 μm. **(E,F)** High magnification image showing triple staining of IFN-α (**E**), GFAP, and DAPI (**F**). E is a single staining panel and F is the merge of all 3 images. Arrows indicate IFN-α-labeled vesicles in remote astrocyte processes. Scales, 10 μm.

Next, we examined IFN-α expression in primary cultures of astrocytes. Immunocytochemistry showed marked IFN-α expression in astrocytes (Fig. 6A). We used the nuclei marker DAPI to stain cells in the cultures, and our data indicated that almost all cells (DAPI+) in cultures expressed GFAP, suggesting that our astrocyte cultures were not contaminated by other cell types (Fig. 6B–D). We found that all IFN-α-positive cells expressed GFAP. Interestingly, IFN-α was observed in vesicles of cytoplasm and remote processes of astrocytes (Fig. 6E,F).

### Exogenous and endogenous IFN-α inhibits pain

**Figure 7:**
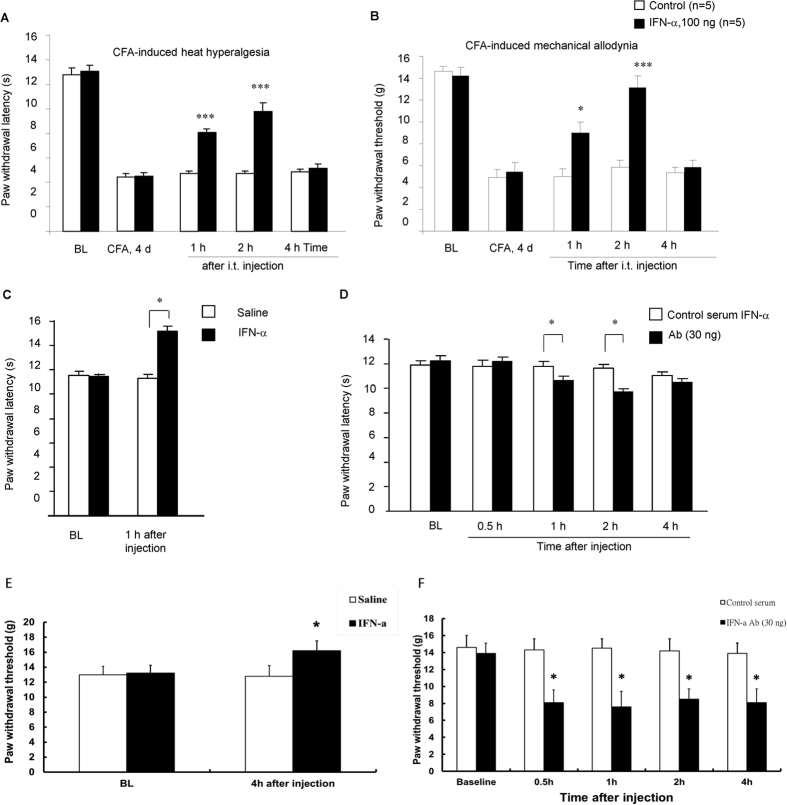
IFN-α is an endogenous pain inhibitor. **(A,B)** Intrathecal injection of IFN-α (100 ng) on CFA day 4 reduces CFA-induced heat hyperalgesia (A) and mechanical allodynia. *P < 0.05; ***P < 0.001, compared to corresponding saline control, *n* = 5 rats/group. (**C**) Intrathecal IFN-α raises paw withdrawal latency in naive rats. *P < 0.05, compared to saline control, *n* = 6 rats/group. (**D**) Intrathecal IFN-α neutralizing antibody (30 ng) decreases paw withdrawal latency in naive rats. *P < 0.05, compared to control serum (30 ng), *n* = 8 rats/group. (**E**) Intrathecal IFN-α decreases paw withdrawal threshold and induces heat hyperalgesia in naive rats. *P < 0.05, compared to control serum (30 ng), *n* = 5 rats/group. (**F**) Intrathecal IFN-α neutralizing antibody (30 ng) decreases paw withdrawal threshold and induces mechanical allodynia in naive rats. *P < 0.05, compared to control serum (30 ng), *n* = 5 rats/group. The data were compared by student’s t-test. All data were mean ± S.E.M.

To determine whether spinal IFN-α would regulate pain sensitivity, we induced inflammatory pain by intraplantar injection of complete Freund’s adjuvant (CFA). Four days after CFA injection, inflamed rats exhibited robust heat hyperalgesia, a reduction in paw withdrawal latency (Fig. 7A), and mechanical allodynia, a reduction in paw withdrawal threshold (Fig. 7B). Intrathecal (i.t.) administration of IFN-α (100 ng) significantly reduced CFA-induced heat hyperalgesia (Fig. 7A) and mechanical allodynia (Fig. 7B) one and two hours after the injection (P < 0.01, n = 5). Intrathecal IFN-α (100 ng) also increased paw withdrawal latency and paw withdrawal threshold in naïve animals (Fig. 7C,E), indicating an analgesic action of IFN-α.

To determine the role of endogenous IFN-α in pain control, we blocked the action of IFN-α with a IFN-α neutralizing antibody. Notably, intrathecal injection of the neutralizing antibody (30 ng) decreased paw withdrawal latency in naïve rats at one and two hours (P < 0.05, n = 8, Fig. 7D) and paw withdrawal threshold for 4 hours after the injection (P < 0.05, n = 5, Fig. 7F), suggesting an induction of heat hyperalgesia and mechanical allodynia by the neutralizing antibody.

## Discussion

Most studies about IFNs focus on their immune functions and transcriptional regulation. The IFN receptors are ubiquitously distributed in various cell types such as immune and endocrine cells^3^. The IFN receptors have extracellular ligand-binding domain and intracellular kinase domain and are activated following ligand-induced dimerization. Interferons modulate gene expression via a simple, direct signaling pathway involving Janus tyrosine kinases (JAK) and signal transducers and activators of transcription (STAT)^1^,^2^. Binding of the IFNs to their cell surface receptors results in a complex cellular response, leading to transcription of a large number of IFN-induced genes^1^.

We made several novel observations in this study. First, type-I IFN receptor (IFN-α/β receptor) was expressed by primary afferent terminals in the superficial dorsal horn. Second, IFN-α inhibited excitatory synaptic transmission and nociceptive transmission in the spinal cord. Third, IFN-α was primarily expressed by astrocytes in the spinal cord. Finally, intrathecal administration of IFN-α elicited analgesic effects. Our findings suggest a new form of neuronal-glial interaction in the spinal cord by which IFN-α, produced by astrocytes, inhibits nociceptive synaptic transmission.

It is striking that type-I and type-II IFN play opposite role in pain regulation at the spinal cord level. As a potent proinflammatory cytokine, the pronociceptive effects of IFN-γ (type-II IFN) are well documented. Intrathecal injection of IFN-γ produces sustained hyperalgesia and allodynia^20^. IFN-γ also enhances excitatory synaptic transmission in the spinal cord^21^. In contrast, we demonstrated an antinociceptive role of IFN-α at the spinal cord level. First, intrathecal administration of IFN-α elicited anti-hyperalgesic and anti-allodynic effect in inflamed animals, and CFA-induced heat hyperalgesia and mechanical allodynia were suppressed by intrathecal IFN-α. Second, intrathecal IFN-α also induced analgesia in naïve animals by increasing baseline paw withdrawal latency. Third, intrathecal administration of an IFN-α neutralizing antibody in naïve animals elicited heat hyperalgesia and mechanical allodynia by decreasing baseline paw withdrawal latency and paw withdrawal threshold. Thus, IFN-α is produced in the spinal cord in normal conditions and serves as a tonic inhibitor of pain. IFN-α may play an important role in maintaining homeostasis of the spinal cord pain circuit. In parallel, a previous study showed that unilateral microinjection of IFN-α into the thalamic nucleus submedius increased paw withdrawal latency, and this analgesic effect was reversed by the non-selective opioid receptor antagonist naloxone and μ selective opioid receptor antagonist beta-FNA^37^. Thus, both spinal and supraspinal IFN-α produces analgesia.

Of interest previous studies suggested that the structure and function of IFN-α are similar to that of endorphin, an opioid peptide. IFN-α has been shown to bind to opioid receptor^38^,^39^. There may even exist distinct domains in the IFN-α molecule, which mediate immune and analgesic effects of IFN-α, respectively^40^. Indeed, both Type-I IFN receptors (IFN-α/βR) and mu opioid receptors are expressed in small-sized DRG neurons and central terminal of primary afferents (Figs 1 and 2)^41^. Whether there is an interaction between these two receptors in the spinal cord for mediating IFN-α’s antinociceptive effect remains to be investigated.

In support of our behavioral studies, we have provided several lines of compelling evidence demonstrating how IFN-α inhibits synaptic transmission and nociception in the spinal cord. First, we found that Type-I IFN receptors (IFN-α/βR) are expressed in neurons, especially in small-size DRG neurons and their central terminals in the superficial dorsal horn. This unique localization of IFN-α/βR in spinal cord presynaptic terminals points to a presynaptic role of IFN-α in regulating neurotransmitter release. Despite this prominent IFN-α/βR expression of in neurons and axons, we should not rule out that this receptor may also be expressed in non-neuronal cells especially in pathological conditions. Second, our patch clamp recording data showed that IFN-α rapidly (within minutes) inhibited the excitatory synaptic transmission (sEPSC) in the dorsal horn (Fig. 3). Since the frequency change of sEPSC is a result of glutamate release, we propose that IFN-α could reduce spinal cord synaptic transmission by inhibiting glutamate release from primary afferent terminals in the spinal cord. Third, our immunohistochemical data showed that IFN-α suppressed capsaicin-induced NK-1 internalization in superficial dorsal horn neurons (Fig. 4A), suggesting that IFN-α can further inhibit substance P release following C-fiber activation. Fourth, our immunohistochemical data revealed that IFN-α also rapidly inhibited capsaicin-induced ERK phopshorylation in superficial dorsal horn neurons (Fig. 4B). Since (i) NK-1 internalization and ERK phosphorylation are reliable markers of nociception at the spinal cord level^29^,^31^ and (ii) ERK phosphorylation after C-fiber activation is a result of glutamate and substance P release^34^, we conclude that IFN-α inhibits spinal cord nociception by inhibiting the release glutamate and substance P from primary afferents.

Accumulating evidence suggests that spinal cord microglia and astrocytes play an important role in the generation of pain hypersensitivity after various tissue and nerve injuries^11^,^13^,^14^,^15^,^16^,^42^. Activated microglia and astrocytes produce proinflammatory cytokines (e.g., TNF-α and IL-1β), chemokines (e.g., MCP-1), and growth factors (e.g., BDNF) to enhance pain^43^,^44^,^45^. The glia-produced pro-inflammatory cytokines and chemokines can powerfully regulate synaptic transmission in spinal cord nociceptive neurons^22^,^45^,^46^,^47^. Thus, several recent reviews have highlighted neuronal-glial interaction as an important mechanism for the genesis of chronic pain^12^,^17^,^48^,^49^,^50^,^51^. In particular, astrocyte-produced IFN-γ might mediate spinal neuronal-glial interactions to drive neuropathic pain, by inducing microglial activation and enhancing synaptic transmission^8^,^20^,^21^.

However, glial “activation” and neural-glial interactions not only lead to enhanced pain states, but may also result in reduced pain states, by producing anti-inflammatory cytokines such as IL-10 from glial cells to inhibit neuronal activity^12^,^52^. Further, activation of cannabinoid receptor type 2 has been shown to induce a microglial anti-inflammatory phenotype via activation of MAP kinase phosphatases, ERK dephosphorylation, and reduced production of proinflammatory mediators^53^. A recent study proposed that chronic inflammation could decrease spinal microglial GRK2, which prevents silencing of microglia/macrophage activity and thereby contributes to prolonged hyperalgesia^54^.

We revealed a new form of neuronal-glial interaction in the spinal cord that can negatively regulate pain sensitivity. We found that IFN-α is primarily expressed by astrocytes in the spinal cord (Fig. 5). Interestingly, IFN-α was observed in vesicles of remote processes of astrocytes (Fig. 6E,F), suggesting that IFN-α is transported to distal processes of astrocytes. Because synapses are enwrapped by astrocytic processes^55^, IFN-α released from remote astrocytic processes that have close contacts with synapses could effectively modulate synaptic transmission, thereby, blocking the flow nociceptive signal from the spinal cord to the brain. In this study, IFN-α was also found to be expressed in few microglial cells (Fig. 5G). Despite a prominent IFN-α expression of in astrocytes, we should not rule out that IFN-α may also be expressed in other cell types in the spinal cord, such as microglia and neurons, in pathological conditions.

In summary, our findings demonstrated that IFN-α is an endogenous pain inhibitor and mediates a novel type of neuronal-glial interaction. Thus, astrocytes-produced IFN-α binds to IFN-α/β receptor expressed by primary afferent terminals to inhibit the release of glutamate and substance P, leading to an inhibition of pain transmission in the spinal cord. Methods of boosting IFN-α release may open a new avenue for pain management. Notably, IFN-α may produce neurotoxicity while IFN-β has neuroprotective effects^56^,^57^. It remains to be investigated if IFN-β, a very close family member of IFN-α (both belong to Type I Interferon), plays the same role in pain regulation.

## Methods

### Animals

Most experiments were performed on adult Sprague-Dawley rats (200–240g, male, purchased from Charles River). Some electrophysiology experiment was conducted in transgenic C57BL/6 mice (5 weeks). These mice express tdTomato fluorescence in somatostatin (SOM^+^) neurons, after *Som*-Cre mice were crossed with tdTomato Cre-reporter mice (Rosa26-floxed stop tdTomato mice), both from Jackson Laboratory, to generate conditional transgenic mice that express tdTomato in SOM^+^ neurons. The animal procedures performed in this study were approved by the Animal Care Committee of Harvard Medical School and Duke University Medical Center. All animals were used under Harvard Medical School and Duke University Medical Center Animal Care institutional guidelines. To produce persistent inflammatory pain, complete Freund’s adjuvant (100 μl, Sigma) was injected into a hindpaw. Intraplantar capsaicin injection (75 μg in 25 μl) was also made to activate C-fibers for acute inflammatory pain. Animals were housed in a 12 h light/dark room with access to food and water *ad libitum*.

### Drugs and administration

We purchased IFN-α (rat and mouse) and IFN-α neutralizing antibody from R & D System. We did pilot experiments to determine the doses of IFN-α in relieving CFA-induced-mechanical allodynia. The doses of 10 and 25 ng were not effective, but the dose of 100 ng was effective in relieving mechanical allodynia. The dose of 15 ng anti-IFN-α was not effective, but the dose of 30 ng was effective in inducing mechanical allodynia in naïve rats. IFN-α (100 ng) was intrathecally injected (20 μl) into naïve rats or inflamed rats 4 days after CFA inflammation. The IFN-α (30 ng) neutralizing antibody was intrathecally injected (20 μl) into naïve rats. For intrathecal injection, a lumbar puncture was made at L5-L6 level with a 30 gauge needle under a brief anesthesia with isoflurane^58^.

### Immunohistochemistry

Animals were terminally anesthetized with isoflurane and perfused through the ascending aorta with saline followed by 4% paraformaldehyde with 1.5% picric acid in 0.16 M PB. After the perfusion the L4-L5 spinal cord segments and DRGs were collected and postfixed in the same fixative overnight. Spinal cord sections (30 μm, free-floating sections) and DRG sections (20 μm) were cut in a cryostat and processed for immunofluorescence. Tissue sections were blocked with 2% goat serum, and incubated over night at 4 °C with the following primary antibodies: IFN-α/βRβ (rabbit, 1:500, Santa Cruz, sc-30015, M-300), IFN-α antibody (rabbit, R&D, 1:1000), GFAP antibody (mouse, 1:5000, Chemicon), OX-42 antibody (mouse, 1:5000, Serotec), NeuN antibody (mouse, 1:5000, Chemicon), CGRP antibody (chicken, 1:2000, Neuromics), substance P antibody (guinea pig, 1:500, Neuromics), NF-200 antibody (mouse, 1:2000, Sigma), NK-1 antibody (rabbit, 1:2000; Neuromics) and phosphorylated ERK antibody (pERK1/2, rabbit, Cell Signaling, 1:500), as well as FITC-conjugated IB4 (1:200, Sigma). The sections were then incubated for 1 h at room temperature with Cy3- or FITC- conjugated secondary antibodies (1:300, Jackson immunolab). For double immunofluorescence, sections were incubated with a mixture of polyclonal and monoclonal primary antibodies followed by a mixture of FITC- and CY3-congugated secondary antibodies. The specificity of immunostaining and antibodies was tested by (1) omission of the primary antibody and (2) absorption of antibodies with respective peptide antigens. To quantify NK-1 immunoreactive cells, 5 free-floating spinal cord sections were randomly selected, and NK-1 receptor internalization in the superficial dorsal horn was determined by the number of labeled endosome-like structures; and those neurons with >15 labeled endosomes were regarded as positive for NK-1 internalization^30^.

For pERK immunostaining and quantification in spinal cord slices, spinal cord slices (600 μm) were fixed in fresh 4% paraformaldehyde for more than 2 hours and transferred to 20% sucrose solution overnight. The slices were embedded in OCT and cut in a cryostat, and 5 non-adjacent sections (20 μm) were selected for pERK immunostaining with pERK antibody (rabbit, Cell Signaling, 1:500), as described above. After the staining, the number of pERK immunoreactive cells in the superficial dorsal horn (laminae I-II) was counted from 5 sections/slice, as previous reported^34^.

### Astrocyte cultures and immunocytochemistry

Astroglial cultures were prepared from cerebral cortexes of neonatal rats (Sprague-Dawley) as we previously described^45^. The meninges were removed from cortexes in Hank’s buffer. Cells were plated at 2.5 × 10^5^/ml in a medium containing 15% FBS in low glucose DMEM and maintained for 2 weeks. The medium was replaced twice a week first with 15% FBS, then with 10% FBS, finally with 10% horse serum. Three days before stimulation, 0.15 mM dibutyryl cAMP (Sigma) was added to induce differentiation of astrocytes. For immunocytochemistry, cultures were fixed with 4% paraformaldehyde for 30 min and processed for double immunofluorescence with a mixture of IFN-α antibody (rabbit, R & D, 1:1000) and GFAP antibody (mouse, 1:5000, Chemicon). The cultures were finally staining with DAPI (Sigma, 1:5000) for 5 min to label all cell nuclei in cultures.

### Spinal slice preparation and pERK immunostaining

A portion of the lumbar spinal cord (L4-L5) was removed from adult rats (200 g) under urethane anesthesia (1.5–2.0 g/kg, i.p.) and kept in pre-oxygenated ice-cold Krebs solution. Transverse slices (600 μm) were cut on a vibrating microslicer. The slices were perfused with Kreb’s solution (10 ml/min) for 3 hours prior to experiment^22^. Some spinal cord slices were used for pERK immunostaining. The slices were stimulated with capsaicin (3 mM, 5 min) or pretreated with IFN-α 10 min before capsaicin stimulation. The slices were then fixed with 4% paraformaldehyde for 1 hour, and cut into thin sections (15 μm) in cryostat for immunohistochemistry using pERK antibody (rabbit, Cell Signaling, 1:500).

### Patch clamp recordings in spinal slices of rats and mice

The whole cell patch-clamp recordings were made from lamina IIo neurons in voltage clamp mode as previously described^22^,^59^. After establishing the whole-cell configuration, neurons were held their holding potentials at −70 mV for recording spontaneous excitatory postsynaptic current (sEPSC). Patch clamp recordings were also conducted in Som+ neurons in lamina IIo of *Som* transgenic mice with tdTomato Cre-reporter. The resistance of a typical patch pipette is 5–10 MΩ, when filled with the internal solution that contains (in mM): potassium gluconate 135, KCl 5, CaCl_2_ 0.5, MgCl_2_ 2, EGTA 5, HEPES 5, ATP-Mg 5 for the recording of sEPSC. Membrane currents were amplified with an Axopatch 200A amplifier (Axon Instruments) in voltage-clamp mode. Signals were filtered at 2 kHz and digitized at 5 kHz. Data were stored with a personal computer using pCLAMP 6 software and analyzed with Axograph 4.0 (Axon Instruments).

### Behavioral analysis in rats

Animals were habituated to the testing environment daily for at least two days before baseline testing. For testing mechanical sensitivity, animals were put in boxes on an elevated metal mesh floor and allowed 30 min for habituation before examination. The plantar surface of each hindpaw was stimulated with a series of von Frey hairs with logarithmically incrementing stiffness (Stoelting), presented perpendicular to the plantar surface (3–5 seconds for each hair). The 50% paw withdrawal threshold was determined using Dixon’s up-down method. Heat sensitivity was tested by radiant heat using Hargreaves apparatus (Stoelting) and the data were presented as paw withdrawal latency (PWL). The radiant heat intensity was adjusted so that basal PWL before inflammation is between 10–12 seconds, with a cut-off of 20 seconds to prevent tissue damage.

### Statistics

All the Data were expressed as mean ± S.E.M. Differences between groups were compared using student’s t-test or ANOVA followed by Fisher’s PLSD posthoc test. For electrophysiology, the difference was compared by paired t-test (two-tailed). The criterion for statistical significance was P < 0.05.

## Additional Information

**How to cite this article**: Liu, C.-C. *et al.* Interferon alpha inhibits spinal cord synaptic and nociceptive transmission via neuronal-glial interactions. *Sci. Rep.* **6**, 34356; doi: 10.1038/srep34356 (2016).
